# RU28318, an Aldosterone Antagonist, in Combination with an ACE Inhibitor and Angiotensin Receptor Blocker Attenuates Cardiac Dysfunction in Diabetes

**DOI:** 10.1155/2013/427693

**Published:** 2013-08-27

**Authors:** Ibrahim F. Benter, Fawzi Babiker, Ibrahim Al-Rashdan, Mariam Yousif, Saghir Akhtar

**Affiliations:** ^1^Department of Pharmacology & Toxicology, Faculty of Medicine, Kuwait University, P.O. Box 24923, 13110 Safat, Kuwait; ^2^Department of Physiology, Faculty of Medicine, Kuwait University, P.O. Box 24923, 13110 Safat, Kuwait; ^3^Department of Medicine, Faculty of Medicine, Kuwait University, P.O. Box 24923, 13110 Safat, Kuwait

## Abstract

*Aims*. We evaluated the effects of RU28318 (RU), a selective mineralocorticoid receptor (MR) antagonist, Captopril (Capt), an angiotensin converting enzyme inhibitor, and Losartan (Los), an angiotensin receptor blocker, alone or in combination with ischemia/reperfusion- (I/R-) induced cardiac dysfunction in hearts obtained from normal and diabetic rats. *Methods*. Isolated hearts were perfused for 30 min and then subjected to 30 min of global ischemia (I) followed by a period of 30 min of reperfusion (R). Drugs were administered for 30 min either before or after ischemia. Drug regimens tested were RU, Capt, Los, RU + Capt, RU + Los, Capt + Los, and RU + Capt + Los (Triple). Recovery of cardiac hemodynamics was evaluated. *Results*. Recovery of cardiac function was up to 5-fold worse in hearts obtained from diabetic animals compared to controls. Treatment with RU was generally better in preventing or reversing ischemia-induced cardiac dysfunction in normal hearts compared to treatment with Capt or Los alone. In diabetic hearts, RU was generally similarly effective as Capt or Los treatment. *Conclusions*. RU treatment locally might be considered as an effective therapy or preventative measure in cardiac I/R injury. Importantly, RU was the most effective at improving −*dP*/*dt* (a measure of diastolic function) when administered to diabetic hearts after ischemia.

## 1. Introduction

In addition to the circulatory renin-angiotensin-aldosterone system (RAAS), there is now a significant body of evidence supporting the concept of a “local tissue or cellular RAAS” that has important roles in the pathology of cardiovascular diseases [1]. The local production of aldosterone and the discovery of mineralocorticoid receptor (MR) expression in the heart have led to a greater understanding of the role of aldosterone/mineralocorticoid receptor activation in the cardiovascular diseases, including hypertension and heart failure [2, 3].

Aldosterone activates its mineralocorticoid receptor (MR) in the nondiabetic heart and can cause structural and electrical remodelling, fibrosis, oxidative stress, inflammation, and arrhythmias [1, 4–7]. MR antagonists have shown significant benefit in patients with left ventricular dysfunction and myocardial infarction [8]. For example, the recent Eplerenone in Mild Patients Hospitalization And Survival Study in Heart Failure (EMPHASIS-HF) study has shown that eplerenone, an MR antagonist, has beneficial effects in patients with moderate heart failure (NYHA class II) [9]. However, beneficial effects of MR blockade in pathological states such as diabetes are unclear. 

It is well established that signaling network alterations in diabetes are such that potential therapies will need to be tailored for this pathological state [10–12]. Inhibitors of the renin-angiotensin-aldosterone system (RAAS), such as angiotensin converting enzyme inhibitors (ACEIs) and angiotensin type-1 receptor (AT_1_) blockers (ARBs), have been shown to protect against hypertension and/or diabetes-induced end-organ damage [13, 14]. However, therapy with ACEIs or ARBs has certain limitations [15]. For example, in a group of 99 patients with severe heart failure, Van De Wal et al. [16] demonstrated that 45% had elevated plasma angiotensin II (Ang II) levels independent of serum ACE activity despite long-term ACE inhibitor use. 

ACEI and/or ARBs do not completely block end-organ damage in diabetes and/or hypertension, and clinical trials of ACEI and ARBs in combination have generally shown that they do not offer added benefits but rather lead to greater adverse effects such as electrolyte imbalance and renal complications [1, 15]. In addition, inhibition of Ang II does not reliably suppress aldosterone production, with the aldosterone escape phenomenon occurring in up to 40% of patients with heart failure [17, 18]. Thus, the need for additional RAAS inhibition in these individuals would be a logical consequence such as combinations of ACEI and/or ARBs with MR antagonist. The Randomized Aldactone Evaluation Study (RALES) and the Eplerenone Postacute Myocardial Infarction Heart Failure Efficacy and Survival Study (EPHESUS) suggested that an MR antagonist on top of an ACEI or an ARB can reduce mortality in patients with severe congestive heart failure and left ventricular dysfunction after MI [15, 18]. Thus, there is an important need to identify the best possible treatment option for normal and diabetic patients with cardiac dysfunction [19]. Important issues that still need to be addressed include identifying the optimal combination of drugs to use and their timing, either administration before or after ischemic injury, in both normal and diabetic states [20]. Hence, the aim of the present study was to characterize and compare the effects of RU28318 (RU, a selective MR antagonist), Captopril (Capt, an ACEI), and Losartan (Los, an ARB), alone or in double and Triple therapy combinations administered either before or after ischemia on ischemia/reperfusion- (I/R-) induced cardiac dysfunction in isolated hearts obtained from normal and diabetic rats.

## 2. Methods

### 2.1. Experimental Procedures

 17-week-old male Wistar rats were divided into 2 groups (*n* = 6 per studied group). Group 1 was control animals and Group 2 was streptozotocin- (STZ-) treated diabetic animals. All animal experiments in this study were approved by the Research Administration at Kuwait University and conformed to their ethics guidelines for the care and use of laboratory animals that are based on those published by the US National Institute of Health (NIH publication no. 85-23, revised 1985). 

### 2.2. Induction of Diabetes

 Diabetes was induced by a single intraperitoneal injection of 55 mg/kg body weight STZ. Basal glucose levels were determined prior to STZ injection and 48 h after STZ injection. Rats with a blood glucose concentration above 250 mg/dL were declared diabetic and any not meeting this criterion were excluded from the study. The animals' diabetic state was re-assessed after 4 weeks just before sacrificing the animals.

### 2.3. Heart Perfusion

 Rats were anesthetized with Intraval Sodium (40 mg/kg body weight), and hearts were rapidly removed after intravenous heparinization (1000 U/kg body weight). The excised hearts were immediately mounted on the Langendorff perfusion assembly (Hugo Sachs Electronics, Freiburg, Germany) and were perfused initially with a constant pressure perfusion of 50 mmHg with the oxygenated (95% O_2_ + 5% CO_2_) Krebs-Henselit buffer (37°C) of the following composition (in mM): NaCl 117; KCl 4.39; CaCl_2_ 2.5; NaHCO_3_ 20.0; KH_2_PO_4_ 1.21; MgCl_2_·6H_2_O 1.2; glucose 12.0; osmolarity 300 mOsm/L, pH 7.35. A water-filled balloon was introduced into the left ventricle and connected to a Statham pressure transducer (P23Db) and balloon volume was adjusted to give the baseline end-diastolic pressure of 5 mmHg. Left ventricular developed pressure (*P*_max⁡_) and its positive and negative derivatives (+*dP*/*dt* and −*dP*/*dt*, resp.) and left ventricular end-diastolic pressure (LVEDP) were continuously monitored. Coronary flow (CF) was measured by means of an electromagnetic flow probe positioned in the inflow tubing immediately above the aortic perfusion cannula. This system permits accurate adjustment of perfusion pressure between 5 and 300 mmHg to an accuracy of ±1 mmHg.

Hearts removed from animals were perfused for 30 min and then subjected to 30 min of global ischemia (I) followed by a period of 30 min of reperfusion (*R*). Drugs were administered for 30 min either during perfusion before ischemia or during reperfusion after ischemia. Drug regimens tested were RU28318 (RU; 10^-5 ^M), Captopril (Capt; 3.6 × 10^-4 ^M), Losartan (Los; 3 × 10^-4 ^M), RU + Capt, RU + Los, Capt + Los, and RU + Capt + Los (Triple). The doses used gave maximal responses in preliminary experiments and were similar to those used in previous studies [21–23]. Post-I/R left ventricular contractility and hemodynamics were recorded. The results are expressed as mean ± SEM. Percent recovery (%*R*) was calculated using the following formula: (reperfusion value)/(baseline value) × 100. The baseline value is the value recorded at 30 min perfusion before exposure to global ischemia.

### 2.4. Statistical Analysis

 Data are presented as mean ± SEM of “*n*” number of experiments. Mean values were compared using analysis of variance followed by Bonferroni's post hoc test. The difference was considered to be significant when *P* value is less than 0.05. Computerized statistical analysis was accomplished with SPSS for Windows (V.6.0.1; SPSS Inc. Evanston, IL, USA).

## 3. Results

### 3.1. Blood Glucose

Diabetes induction by STZ injection led to a significant increase in blood glucose concentration. Hyperglycaemia persisted throughout the study period and was 306 ± 18 mg/dL at four weeks in STZ-treated animals compared to 96 ± 4 mg/dL in the nondiabetic (control) animals. There were no significant differences in body (275 ± 17 gm) and heart weights (0.95 ± 0.03 gm) corrected to tibia length in these animals. 

### 3.2. Heart Perfusion Studies

**Figure 1:**
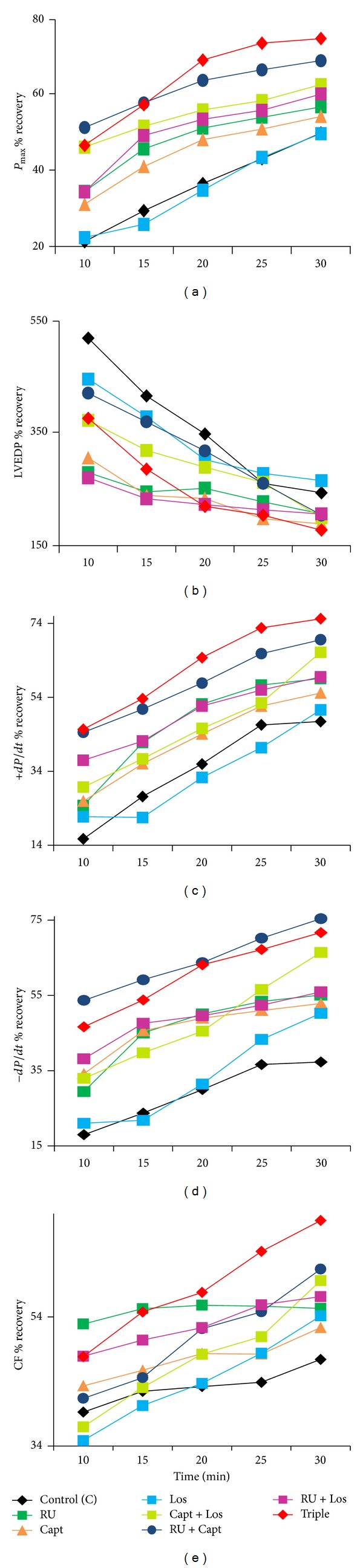
The effect of drugs given before ischemia on % recovery in cardiac function versus reperfusion time for (a) *P*_max⁡_, (b) LVEDP, (c) +*dP*/*dt*, (d) −*dP*/*dt*, and (e) CF in control hearts. RU: RU28318; Capt: Captopril; Los: Losartan; Triple: RU28318 + Captopril + Losartan; *P*_max⁡_: left ventricular developed pressure; LVEDP: left ventricular end-diastolic pressure; +*dP*/*dt*: positive derivative of pressure; −*dP*/*dt*: negative derivative of pressure; CF: Coronary flow; %*R*: % recovery: (reperfusion/baseline) × 100.

**Figure 2:**
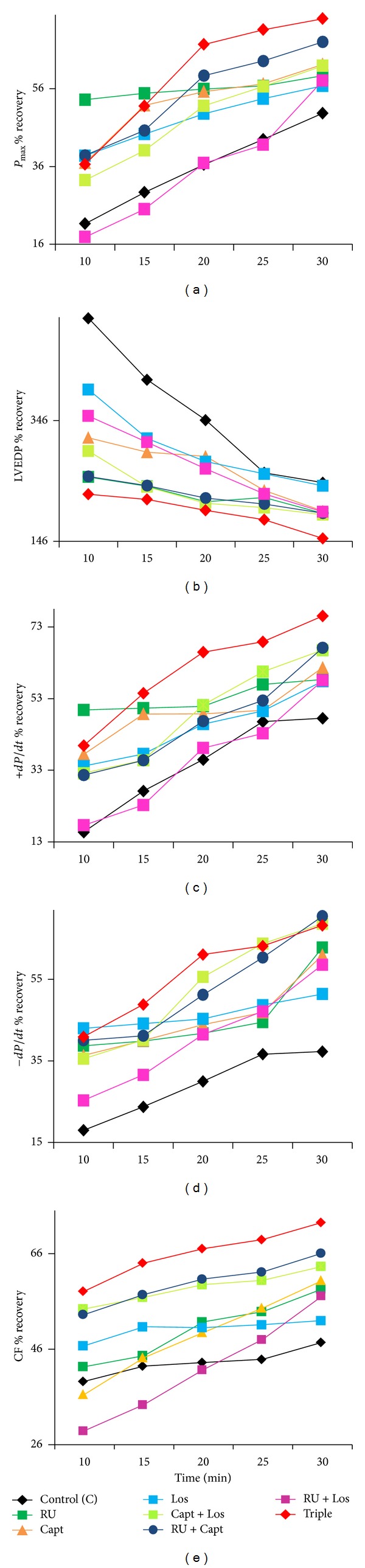
The effect of drugs given after ischemia on % recovery in cardiac function versus reperfusion time for (a) *P*_max⁡_, (b) LVEDP, (c) +*dP*/*dt*, (d) −*dP*/*dt*, and (e) CF in control hearts. RUL: RU28318; Capt: Captopril; Los: Losartan; Triple: RU28318 + Captopril + Losartan; *P*_max⁡_: left ventricular developed pressure; LVEDP: left ventricular end-diastolic pressure; +*dP*/*dt*: positive derivative of pressure; −*dP*/*dt*: negative derivative of pressure; CF: coronary flow; %*R*: % recovery: (reperfusion/baseline) × 100.

**Figure 3:**
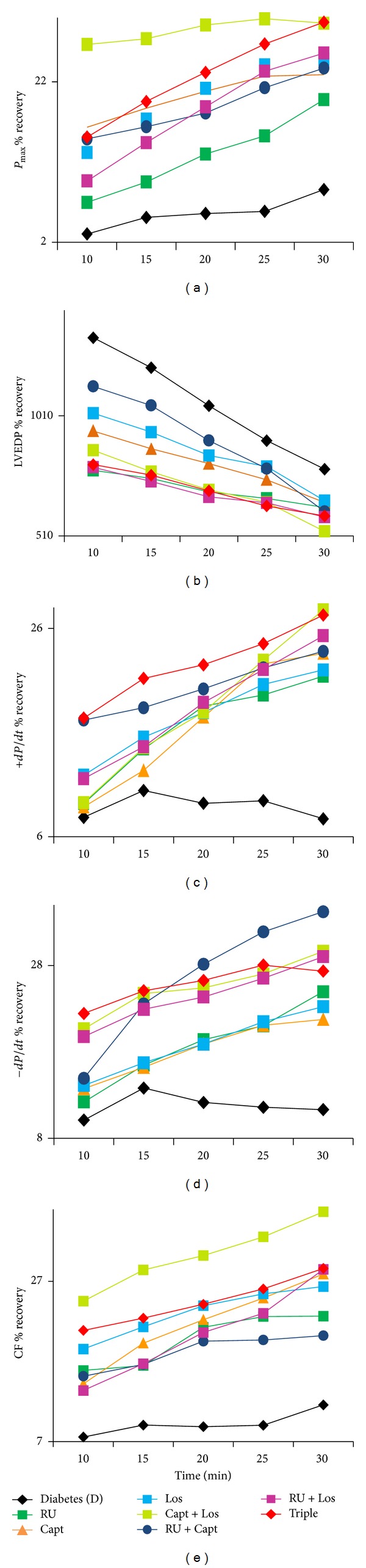
The effect of drugs given before ischemia on % recovery in cardiac function versus reperfusion time for (a) *P*_max⁡_, (b) LVEDP, (c) +*dP*/*dt*, (d) −*dP*/*dt*, and (e) CF in diabetic hearts. RU: RU28318; Capt: Captopril; Los: Losartan; Triple: RU28318 + Captopril + Losartan; *P*_max⁡_: left ventricular developed pressure; LVEDP: left ventricular end-diastolic pressure; +*dP*/*dt*: positive derivative of pressure; −*dP*/*dt*: negative derivative of pressure; CF: coronary flow; %*R*: % recovery: (reperfusion/baseline) × 100.

**Figure 4:**
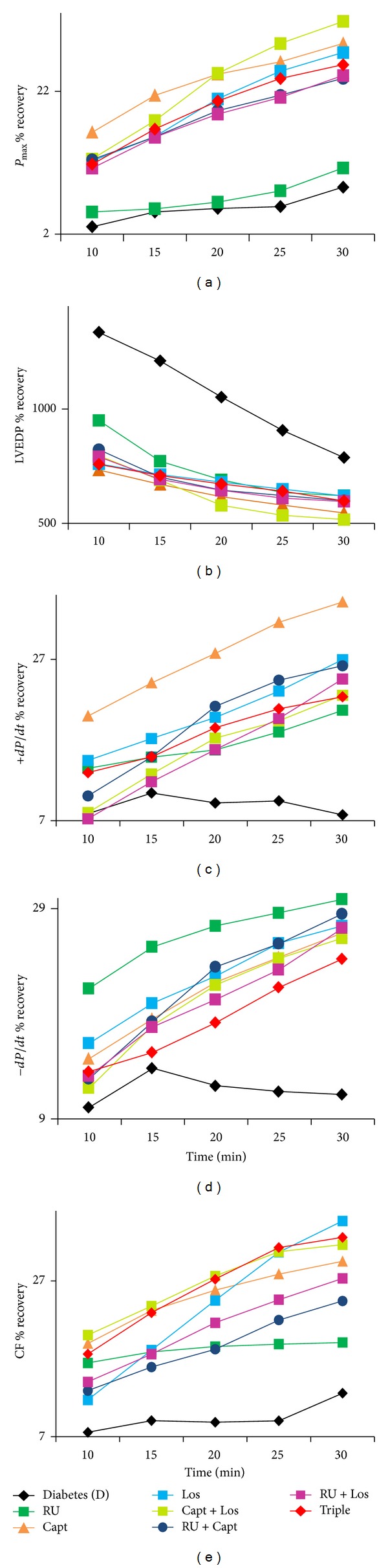
The effect of drugs given after ischemia on % recovery in cardiac function versus reperfusion time for (a) *P*_max⁡_, (b) LVEDP, (c) +*dP*/*dt*, (d) −*dP*/*dt*, and (e) CF in diabetic hearts. RU: RU28318; Capt: Captopril; Los: Losartan; Triple: RU28318 + Captopril + Losartan; *P*_max⁡_: left ventricular developed pressure; LVEDP: left ventricular end-diastolic pressure; +*dP*/*dt*: positive derivative of pressure; −*dP*/*dt*: negative derivative of pressure; CF: coronary flow; %*R*: % recovery: (reperfusion/baseline) × 100.

The effects of various acute drug treatments (RU, Capt, Los, Capt + Los, Capt + RU, and Los + RU, Capt + Los + RU) administered during perfusion (before ischemia) or during reperfusion (after ischemia) on recovery of each cardiac function parameter following I/R in normal and diabetic hearts were recorded. The percent (%) recovery in *P*_max⁡_, LVEDP, +*dP*/*dt*, −*dP*/*dt*, and CF as a function of reperfusion time for nondiabetic control hearts is shown in Figure 1 (before ischemia) and Figure 2 (after ischemia) and for diabetic hearts in Figure 3 (before ischemia), and Figure 4 (after ischemia). In general, drug treatments resulted in a very rapid rate of cardiac function recovery within the first 10 min of reperfusion followed by a more gradual rate of recovery (Figures 1–4). RU is the best single treatment in nondiabetic control hearts when given before ischemia. RU resulted in the most rapid recovery in CF within the first 10 min of reperfusion for CF when given before ischemia and in +*dP*/*dt* and *P*_max⁡_ when administered after ischemia in control hearts (Figures 1 and 2). In diabetic hearts, RU, RU + Los, and Triple therapy similarly gave the most rapid recovery within 10 mins of reperfusion for LVEDP when given before ischemia. Interestingly, Cap + Los therapy given before ischemia in diabetic hearts resulted in a very rapid improvement in *P*_max⁡_ and CF within the first 10 min of reperfusion that was almost the maximal recovery obtained (Figures 3(a) and 3(e)). RU + Capt and Triple therapy given before ischemia to diabetic hearts resulted in the most rapid recovery in +*dP*/*dt*. RU + Los yielded the most rapid recovery in LVEDP when given before ischemia in nondiabetic hearts. Other significantly rapid recoveries in function were observed for +*dP*/*dt* for Capt alone given after ischemia and for −*dP*/*dt* when RU was given after ischemia in diabetic hearts (Figures 4(c) and 4(d)). 

#### 3.2.1. The Effect of Drug Treatments on *P*_max⁡_ in Control and Diabetic Hearts

**Figure 5:**
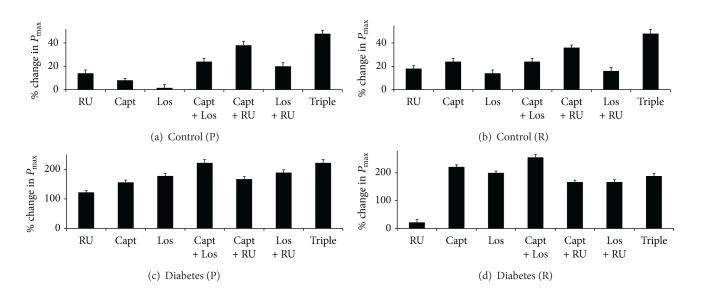
A comparison of the % change in left ventricular developed pressure (*P*_max⁡_) in control and diabetic hearts following acute treatment with various drug regimens. Drugs were given to control hearts during perfusion (a) or reperfusion (b) and to diabetic hearts during perfusion (c) or reperfusion (d). The percent change in parameter is calculated relative to the % recovery seen in the respective nondiabetic or diabetic controls. RU = RU28318; Capt: Captopril; Los: Losartan; Triple: RU28318 + Captopril + Losartan.

In untreated controls, % recovery (%*R*) of *P*_max⁡_ at the end of the 30 min reperfusion period was around 50%. Administration of RU either before ischemia (during perfusion) or after ischemia (during reperfusion) led to a similar but significant improvement in *P*_max⁡_ of between 14 and 18% (see Figure 5(a)). Of the individual (single) drug treatments administered before ischemia, the improvement (% change in function relative to untreated controls) in *P*_max⁡_ with RU was the highest compared to Capt alone (8%), whereas Los showed no significant improvement (Figure 5(a)). However, when single drugs were administered after ischemia, the % improvement in *P*_max⁡_ was the highest for Capt at around 25%, and again the least improvement was seen with Los at around 14%. When drugs were administered as combinations, Triple therapy (Capt + Los + RU) yielded the best recovery in *P*_max⁡_ of about 74%—an improvement over controls of 48%—irrespective of whether it was administered before or after ischemia (Figure 5). Of the double combinations, only Capt + RU was significantly (*P* < 0.05) better over either of the single agents alone when administered either before or after ischemia (Figure 5). 

Recovery in *P*_max⁡_ of diabetic hearts (%*R* of 9 ± 3 mmHg) from I/R was significantly (*P* < 0.05) impaired compared to nondiabetic controls (%*R* of 50 ± 2 mmHg). Administration of all drug treatments to diabetic hearts before ischemia led to significant improvement in *P*_max⁡_ of between 122 and 222% (see Figure 5(c)). Again in general, the Triple therapy appeared to be better than double therapies which appeared to be better than single therapies with some notable exceptions. Capt + Los was equally as effective as the Triple combination, and Capt + RU double combination was not significantly (*P* < 0.05) better than any of the single therapies (Figure 5). However, when single drugs were administered after ischemia in diabetic hearts, interestingly, there was no significant improvement in *P*_max⁡_ with RU (Figure 5(d)). This is in marked contrast to what was observed in the nondiabetic controls (Figures 5(a) and 5(b)). However, all other drug treatments gave comparable improvements in *P*_max⁡_ with Capt + Los being the best treatment option (Figures 5(c) and 5(d)).

#### 3.2.2. The Effect of Drug Treatments on LVEDP in Control and Diabetic Hearts

**Figure 6:**
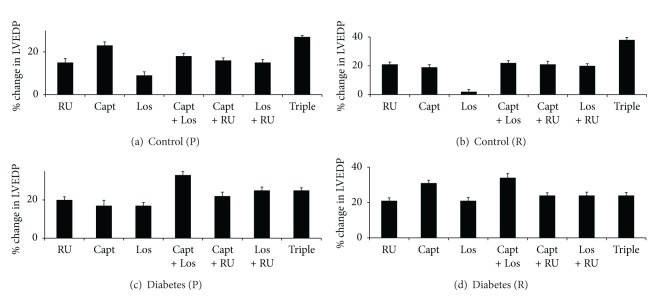
A comparison of the % change in left ventricular end-diastolic pressure (LVEDP) in control and diabetic hearts following acute treatment with various drug regimens. Drugs were given to control hearts during perfusion (a) or reperfusion (b) and to diabetic hearts during perfusion (c) or reperfusion (d). The percent change in parameter is calculated relative to the % recovery seen in the respective nondiabetic or diabetic controls. RU: RU28318; Capt: Captopril; Los: Losartan; Triple: RU28318 + Captopril + Losartan.

%*R* in LVEDP of around 250% was observed in nondiabetic controls that could be significantly reduced (*P* < 0.05) (i.e., improved) by all drug treatments (Figures 6(a) and 6(b)). In the case of single therapies for control (nondiabetic) hearts, acute treatment with Capt gave the best improvement in LVEDP when given before ischemia (Figure 6(a)) and was joint best with RU when given after ischemia (Figure 6(b)). Los treatment after ischemia in control hearts yielded minimal improvement in LVEDP. Double therapies gave no advantage in LVEDP improvement over single therapies, whereas Triple therapy (Capt + Los + RU) yielded the best recovery in LVEDP of about of 27% and 38%, respectively, when given before or after ischemia (Figures 6(a) and 6(b)). Capt given before ischemia gave similar improvement of about 23% to the Triple therapy (i.e., % improvement values were not significantly different; *P* < 0.05; see Figure 6(a)).

In diabetes, LVEDP was significantly (*P* < 0.05) elevated compared to nondiabetic control hearts by over 3-fold. Capt + Los double therapy yielded the best improvement of around 30% in LVEDP when given either before or after ischemia (Figures 6(c) and 6(d)). Capt alone was equally effective as Capt + Los when administered after ischemia (Figure 6(d)). In the case of LVEDP, all other therapies gave similar % improvements in function (Figures 6(c) and 6(d)). Interestingly, Los given alone in diabetic hearts was relatively more effective at improving LVEDP than in nondiabetic control hearts (Figures 5(a) and 5(b); Figures 6(c) and 6(d)). In contrast to *P*_max⁡_ where greater improvements in function were observed for diabetic hearts (Figure 5), in the case of LVEDP, drug-induced improvements appeared similar for diabetes and controls when presented as % change (Figure 6). The reasons for this apparent anomaly are that in diabetes LVEDP significantly (*P* < 0.03) increases by about 3-fold, and thus in calculating a % change, the denominator is now a large number and thus yields modest changes as a percentage. However, when considering the actual numerical changes induced by the different drug treatments in terms of %*R* for LVEDP, this suggests that the degree of change induced by drugs was considerably higher (about 2–5 fold) in diabetic hearts compared to control hearts.

#### 3.2.3. The Effect of Drug Treatments on +*dP*/*dt* in Control and Diabetic Hearts

**Figure 7:**
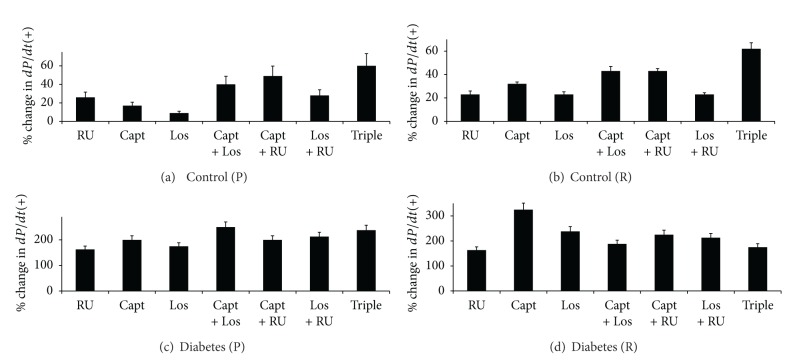
A comparison of the % change in positive derivative of pressure (+*dP*/*dt*) in control and diabetic hearts following acute treatment with various drug regimens. Drugs were given to control hearts during perfusion (a) or reperfusion (b) and to diabetic hearts during perfusion (c) or reperfusion (d). The percent change in parameter is calculated relative to the % recovery seen in the respective nondiabetic or diabetic controls. RU: RU28318; Capt: Captopril; Los: Losartan; Triple: RU28318 + Captopril + Losartan.

In untreated controls, % recovery of +*dP*/*dt* following I/R was around 47% and similar to that observed for *P*_max⁡_ (Figure 5). Drug treatments significantly (*P* < 0.02) improved function when administered either before or after ischemia (Figures 7(a) and 7(b)). In general, triple therapy (around 60% improvement) was better than double therapy which in turn was more effective than single therapies in improving recovery of +*dP*/*dt* (Figures 7(a) and 7(b)). Of the single therapies, LOS appeared to be the least effective in improving +*dP*/*dt* (Figures 7(a) and 7(b)). RU was the most effective when given before ischemia whereas Capt was the best when given after ischemia in improving +*dP*/*dt* (Figures 7(a) and 7(b)).

Diabetes (8 ± 2%) led to about a 6-fold reduction in %*R* for +*dP*/*dt* as compared to controls (47 ± 2%). Drug treatments generally led to marked and significant improvement in function. When given before ischemia, drugs gave similar improvements in function at the end of perfusion, but Triple therapy as well as RU + Capt gave the most rapid improvement within the first 10 min of perfusion. In the case of Capt + Los, approximately 250% improvement was attained which was similar to that obtained with Triple therapy when given before ischemia. When given after ischemia, surprisingly Capt alone (approx 300% improvement) was the best therapy (Figure 7). All drug treatments generally showed greater % improvement in diabetes compared to controls where less than 60% improvement in +*dP*/*dt* was noted (Figure 7).

#### 3.2.4. The Effect of Drug Treatments on −*dP*/*dt* in Control and Diabetic Hearts

**Figure 8:**
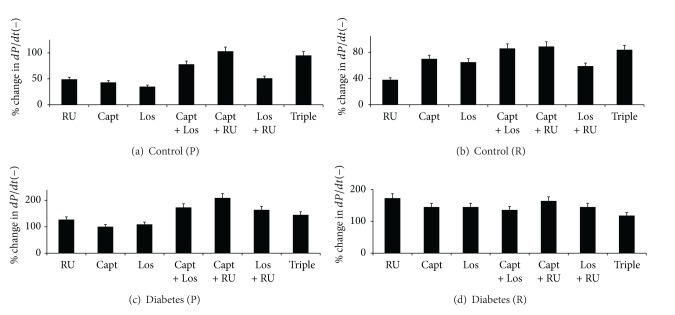
A comparison of the % change in negative derivative of pressure (−*dP*/*dt*) in control and diabetic hearts following acute treatment with various drug regimens. Drugs were given to control hearts during perfusion (a) or reperfusion (b) and to diabetic hearts during perfusion (c) or reperfusion (d). The percent change in parameter is calculated relative to the % recovery seen in the respective nondiabetic or diabetic controls. RUL: RU28318; Capt: Captopril; Los: Losartan; Triple: RU28318 + Captopril + Losartan.

In untreated controls, % recovery (%*R*) of −*dP*/*dt* following I/R was around 37%. Drug treatments significantly (*P* < 0.05) improved −*dP*/*dt* by about 40–100% when administered either before or after ischemia (Figures 8(a) and 8(b)). All the single therapies when administered before ischemia gave similar improvements in recovery of −*dP*/*dt* (Figure 8(a)). With the exception of Los + RU, double therapies were better than single therapies and showed similar effectiveness as the Triple therapy (Figure 8(a)). When drugs were administered after ischemia, with the exception of Los alone which exhibited the least improvement of about 40%, all other treatment regimens were similarly effective exhibiting improvements in the range 60–80% (Figure 8(b)).

Diabetes (11 ± 3%) led to a about a 3.4-fold reduction in %*R* for −*dP*/*dt* as compared to controls (37 ± 2%). Drug treatments in diabetic hearts generally led to marked and significant improvement in function from about 100% to 200% and this was generally higher compared to that seen following drug treatments in control hearts (see Figures 8(c) and 8(d)). All drug treatments were equally effective in improving −*dP*/*dt* when administered after ischemia (Figure 8(c)), whereas combination therapies was more effective than single therapies when administered before ischemia (Figure 8(d)). Administration of Capt + RU before ischemia and RU alone after ischemia were the most effective treatment in improving −*dP*/*dt* in diabetic hearts (Figure 8(c)).

#### 3.2.5. The Effect of Drug Treatments on CF in Control and Diabetic Hearts

**Figure 9:**
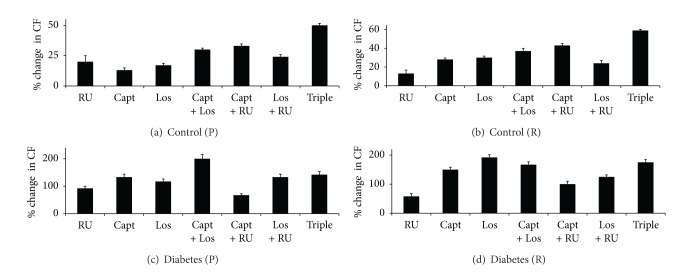
A comparison of the % change in coronary flow (CF) in control and diabetic hearts following acute treatment with various drug regimens. Drugs were given to control hearts during perfusion (a) or reperfusion (b) and to diabetic hearts during perfusion (c) or reperfusion (d). The percent change in parameter is calculated relative to the % recovery seen in the respective nondiabetic or diabetic controls. RU: RU28318; Capt: Captopril; Los: Losartan; Triple: RU28318 + Captopril + Losartan.

In untreated controls, % recovery (%*R*) of CF following I/R was around 43%. Drug treatments significantly (*P* < 0.05) improved function by about 20–60% when administered either before or after ischemia (Figures 9(a) and 9(b)). When drugs were administered before ischemia, RU gave the most rapid recovery within the first 10 mins of reperfusion. Triple therapy was generally better than double therapies which in turn were more effective than single agents (Figure 9(a)). Triple therapy (60% improvement) was also more effective when administered after ischemia, and with the exception of Los alone (around 10% improvement), all other treatments yielded similar improvements in the range 30–40%; see Figures 9(a) and 9(b).

Diabetes (12 ± 3%) led to about a 3.8-fold reduction in %*R* for CF as compared to controls (46 ± 3%). Drug treatments in diabetic hearts generally led to marked and significant improvement in function from about 100% to 200% and this was generally higher (Figures 9(c) and 9(d)), compared to that seen following drug treatments in control hearts (Figures 9(a) and 9(b)). All other drug treatments were equally effective in improving CF when administered before ischemia except for Capt + Los which was the best treatment (about 200% improvement); see Figures 3(c) and 9(c). Given after ischemia, Capt, Los, Capt + Los, and Triple therapy were similarly effective in improving CF in diabetic hearts, whereas RU alone was the least effective (Figure 9(d)).

## 4. Discussion

A major goal of this study was to identify the optimal treatment strategy of RAAS blockade to prevent or treat ischemia-reperfusion injury in normal and diabetic hearts [24–26]. Although the use of ACEI or ARBs has been well studied, the effects of aldosterone antagonism alone or in combination with these other RAAS blockers especially in diabetes are not well understood. 

This study showed that treatment with RU was generally better in preventing or reversing ischemia-induced cardiac dysfunction in normal hearts compared to treatment with an ACEI (Capt) or ARB (Los) alone. In the case of diabetic hearts, RU was generally similarly effective as Capt or Los treatment. Also, dual therapies involving RU were similarly effective as Capt + Los therapy, whereas Triple combination was generally equal to or the most effective strategy in preventing or reversing ischemia-induced cardiac dysfunction in normal or diabetic hearts. For example, irrespective of whether drugs were administered before or after ischemia, combination therapies appeared more effective, whereby triple > double > single therapy in most of the study scenarios (i.e., 18 out of the 20 experimental scenarios of looking at 5 different cardiac parameters studied for both normal and diabetic hearts for when drugs were given either before or after ischemia). In contrast to nondiabetic hearts, Capt + Los dual therapy was the most effective therapy (alongside Triple therapy) in diabetic hearts which is consistent with the known diabetes-induced overactivity of the ACE/Ang II/AT_1_ signaling cascade [16, 27, 28].

In this study, looking at an MR antagonist in diabetic hearts, we show that indeed the diabetic pathology leads to altered cardiac response to treatments with RU as well as ARBs and ACEI. In general, with the exception of *P*_max⁡_ for RU given after ischemia, the relative improvement in cardiac function or effectiveness of RAAS blockers was greater in diabetes than in control hearts. Thus, in nondiabetic hearts, RU alone was highly effective at preventing and treating I/R-induced cardiac dysfunction, whereas in diabetes, in terms of *P*_max⁡_, it was only effective at preventing (i.e., when given before ischemia) I/R-induced cardiac injury. It was ineffective, in terms of *P*_max⁡_, when given after ischemia in diabetes implying a differential role of aldosterone MR signaling in the diabetic pathology. These data suggest that MR signaling is detrimental for all cardiac parameters in nondiabetic hearts during ischemia as well as reperfusion phases, whereas in the diabetic hearts, at least for *P*_max⁡_, it may be detrimental only during the ischemia-induced phase of I/R injury. The reasons for the differential response in *P*_max⁡_ in diabetes are not understood but clearly require further study. Indeed, the effectiveness of drugs generally appeared to be cardiac parameter specific with some drugs being better at improving one cardiac function parameter over others. For example, although RU was ineffective at improving *P*_max⁡_, it was the most effective at improving −*dP*/*dt* when administered to diabetic hearts after ischemia. This may imply that RU may not improve systolic function but may show marked improvements in diastolic function in diabetic hearts when given after ischemia. This assertion is further supported by the fact that the RU significantly (*P* < 0.03) improved LVEDP in diabetic hearts (Figures 3, 4, and 6). RU exhibited greater beneficial effects in LVEDP for diabetic compared to control hearts when considering the actual numerical changes in terms of %*R* for LVEDP.

Although RU-mediated improvement in cardiac function generally appears to be as good as ARBs and ACEIs, differences exist between RU and other drugs as to effectiveness when given before or after ischemia. In nondiabetic hearts, RU was the best or one of the best single agents when given before or after ischemia, whereas in diabetes, it generally provided minimal or least benefit when given after ischemia with the exception of −*dP*/*dt* where RU was the best treatment option. This finding may be clinically relevant as diabetes patients generally present with compromised diastolic function as an early indicator of cardiac dysfunction and is detected in about 75% of asymptomatic diabetic patients [29, 30]. Our results show that RU importantly improves diastolic function (as measured by −*dP*/*dt*) in diabetes especially when given after ischemia and thus early-stage RU treatment may represent a novel strategy for treating diabetes-induced diastolic dysfunction. 

Our study also showed that irrespective of whether drugs were administered before or after ischemia, their effectiveness in recovering cardiac function appeared to be time dependent; typically, drugs rapidly improved cardiac function within the first 10 min of reperfusion followed by a period of steady improvement thereafter up to the 30 min study period (Figures 1–4). However, there were some interesting differences as to which cardiac function parameters were improved during this initial rate of recovery. For example, significantly rapid recoveries in function were observed for +*dP*/*dt* for therapy with Captopril alone when administered after ischemia and for −*dP*/*dt* when RU was given after ischemia in diabetic hearts (Figures 4(c) and 4(d)). These data further imply that in patients with diabetes where diastolic function is often the only early indicator of cardiac dysfunction, these treatments offering rapid improvements in ±*dP*/*dt* should be considered as potential therapeutic options. 

The possible mechanisms by which RU may be acting beneficially in normal and diabetic hearts subjected to I/R are not known but these may include blockade of aldosterone-induced oxidative stress, endothelial dysfunction, and inflammation in the heart and in the coronary vasculature that generally contribute to abnormal calcium homeostasis and cardiac dysfunction. Thus, RU-mediated blockade of these important processes likely also improves calcium handling and overload in hearts subjected to I/R. This assertion is supported by previous findings that aldosterone/MR activation induces cardiomyocyte ionic remodelling by modulating potassium and L-type calcium channel activity [31, 32], T-type calcium channel expression [33], and ryanodine receptor activity [34]. Changes in these calcium handling proteins have been reported to lead to important consequences in the control of calcium signaling, modulation of calcium transients, sarcoplasmic reticulum diastolic leaks, and promotion of rhythm disorders that can be corrected by aldosterone/MR antagonism [35–37]. 

Aldosterone is also known to induce increased Na^+^/H^+^ exchanger-1 (NHE-1) activity via transactivation of the epidermal growth factor receptor (EGFR) and subsequent reactive oxygen species (ROS) formation which is thought to be an important signaling cascade in the genesis of many cardiac pathologies [38]. This may also lead to calcium overload that is detrimental to cardiac function. Hence, another possible mechanism by which MR antagonism with RU may be exerting its beneficial effects is via inhibition of the detrimental cardiac EGFR/NHE-1 signaling network. However, we have recently shown that EGFR signaling via pathways involving ERK1/2, p38 MAP kinase, and Akt/FOXO appears to be an important beneficial survival mechanism, and its inhibition leads to worsening of cardiac function following I/R [39]. Thus, targeting EGFR inhibition with MR antagonists such as RU may have beneficial and detrimental consequences in the heart and thus their net therapeutic advantage will depend on the relative importance or contributions of these different EGFR-driven mechanisms in a given pathological state. Indeed, we have recently shown that by removing the EGFR inhibitory effects of Los, which blocks Ang II-mediated transactivation of EGFR, by coadministering an EGFR ligand, significant improvement in cardiac function over that achieved by Los alone was attained [40]. 

Whether the beneficial effects of RU may be via antagonism of aldosterone-mediated effects in cardiac muscle and/or vasculature is not clear but recent studies suggest the involvement of both. It appears that cardiomyocyte aldosterone/MR participates in the crosstalk between cardiomyocytes and coronary blood vessels, such as increased aldosterone synthesis by the cardiomyocytes resulting in coronary dysfunction [1, 41]. Interestingly, signaling by EGFR, the transactivation target of aldosterone, is known to be elevated in the diabetic vasculature and is detrimental to vascular function [40, 42–44]. Thus, RU-mediated blockade of aldosterone-induced EGFR transactivation may be beneficial in improving diabetes-induced vascular dysfunction in the heart but this requires further study.

In diabetic patients, in addition to cardiac dysfunction, there is also significant risk of renal damage where Ang II blockade is contraindicated as it will lead to attenuation of Ang II-driven glomerular filtration rate (GFR) and renal shutdown particularly in patients with renal artery stenosis. However, RU which does not affect GFR may be particularly useful for such patients. Further, addition of RU as a combination strategy with ACEI and ARBs may allow for a reduction in the dose of the latter agents such that they have minimal disruption on GFR and electrolyte balance whilst retaining their ability to reduce diabetes-induced proteinuria and their beneficial effects on cardiac function as highlighted in the present study.

Importantly, MR antagonists when combined with ACEI but not ARBs have shown significant reduction in total mortality in patients with CHF [45]. But here in I/R injury, we show generally that combination approaches are better where Capt + Los + RU (Triple) therapy is mostly the best treatment option in normal and diabetes. However, combination therapies with ACEI and ARBs are rarely employed in the clinic possibly due to fear over accumulating adverse effects in patients with heart disorders [15, 20, 46]. Whether the inclusion of RU in the highly effective Triple therapies described herein can lead to dose reduction of ACEI and/or ARBs to minimize or eliminate these adverse effects requires further clinical study. 

In diabetic patients, therapies involving aldosterone/MR antagonists may have other additional beneficial effects beyond the cardiovascular system. They may oppose aldosterone-mediated detrimental effects on structural and functional integrity of the pancreatic [beta]-cell resulting from islet cell inflammation and oxidative stress as well as aldosterone-induced insulin resistance [47, 48].

If our findings reported here are reproduced in clinical studies, our study may have important clinical implications in the way these drugs should be administered in cardiac dysfunction. Firstly, our data implies that in normal patients, RU alone could be an effective therapy for prevention of cardiac dysfunction because as a single agent, it yields the best improvements in cardiac function when given before ischemia. Furthermore, for postischemic injury, although RU appears to be an effective therapy, ACEIs and/or ARBs appear to be the drugs of choice for diabetics as they yielded the best improvements in cardiac function when administered after ischemia. We also suggest that MR antagonists, since they act through a non-ACE/Ang II/AT1R pathway, may represent a novel class of RAAS inhibitor that potentially could overcome the limitations observed with the ACEI and ARB combinations of RAAS inhibitors.

Our study by selecting to administer drugs acutely in isolated hearts is advantageous in that it examines the effects of these therapeutic agents directly on the heart and avoids noncardiac contributions of these agents. Furthermore, this study highlights that in addition to the benefits observed by MR antagonists when administered systemically, these agents can also be beneficial when administered locally. Thus, our data implies that in the clinic RU treatment locally might be considered as an effective therapy or preventative measure in cardiac I/R injury for susceptible patients and possibly also preoperatively for patients undergoing aortic cross-clamping or other cardiac surgeries such as cardiopulmonary bypass or coronary artery bypass grafting. Our study also suggests that optimal usage of drug(s) alone or in combination may require their selection based on several criteria including their relative benefit in the normal versus pathological state, whether being considered for prevention or treatment strategy, on the specific cardiac parameter that might need to be improved (e.g., diastolic function in diabetes) and on the optimal rate of cardiac function recovery required for a given condition.
